# Prevalence of osteopathologies in a single center cohort of survivors of childhood primary brain tumor

**DOI:** 10.3389/fped.2022.913343

**Published:** 2022-07-18

**Authors:** Michael M. Schündeln, Sebastian Fritzemeier, Sarah C. Goretzki, Pia K. Hauffa, Martin Munteanu, Cordula Kiewert, Berthold P. Hauffa, Gudrun Fleischhack, Stephan Tippelt, Corinna Grasemann

**Affiliations:** ^1^Pediatric Hematology and Oncology, Department of Pediatrics III, University Hospital Essen, University of Duisburg-Essen, Essen, Germany; ^2^Pediatric Endocrinology and Diabetology, Department of Pediatrics II, University Hospital Essen, University Duisburg-Essen, Essen, Germany; ^3^Department of Pediatric Endocrinology and Diabetology, Caritas Hospital, Bad Mergentheim, Germany; ^4^Department of Pediatrics, Division of Rare Diseases, St. Josef-Hospital Bochum, Ruhr-University Bochum, Bochum, Germany

**Keywords:** childhood primary brain tumors, bone health, childhood malignancies, osteopathologies, survivorship, vitamin D

## Abstract

**Background:**

Childhood primary brain tumors (CPBT) are the second largest group of childhood malignancies and associated with a high risk for endocrine late effects.

**Objective:**

To assess endocrine late effects and their relevance for the development of osteopathologies in survivors.

**Methods:**

This single center cross sectional study investigated data from 102 CPBT survivors with a mean age of 13.0 years and a mean age at diagnosis of 8.7 years. Clinical, biochemical, radiographic, and anamnestic data regarding endocrine and bone health were obtained at study visits. In addition, data regarding tumor stage and therapy was obtained by chart review. An expert opinion was applied to define presence of osteopathologies.

**Results:**

Impaired bone health, defined by at least one pathological screening parameter, was present in 65% of patients. 27.5% were found to have overt osteopathologies per expert opinion. 37.8% displayed a severe vitamin D deficiency (25-OH vitamin D < 10 ng/ml) and 11% a secondary hyperparathyroidism. Patients with osteopathologies had lower 25-OH vitamin D levels compared to patients without osteopathologies. Multiple endocrine late effects were present: diabetes insipidus in 10.8%, aberrant pubertal development in 13.7%, central hypocortisolism in 14.9%, thyroid dysfunction in 23.8% and growth hormone deficiency in 21.8%. A total of 31.3% of survivors displayed any endocrinopathy. Tumors located near hypothalamic structures and patients who received irradiation had a higher likelihood of endocrine morbidity.

**Conclusion:**

This study indicates that endocrine deficiencies are common in pediatric survivors of CPBTs. Osteopathologies are present in this cohort. A prominent effect of hormonal deficiencies on bone health was not detected, possibly because patients were sufficiently treate for their endocrine conditions or indicating resilience of the childhood bone remodeling process. Vitamin D deficiency is frequent and should be treated as recommended.

## Introduction

Childhood primary brain tumors (CPBTs) represent the second largest group of childhood malignancies following acute lymphoblastic leukemia (ALL; 1). CPBTs comprise of a multitude of entities with a broad spectrum of biological characteristics as reviewed by Pollack et al. (2).

As in other childhood malignancies, the treatment outcome of CPBTs has improved markedly in the past decades. The overall 5-year survival is currently reported to be over 74% for all forms of CPBTs (3, 4). However, tumor- and therapy-associated long term morbidities are becoming increasingly apparent in long term survivors (5).

In survivors of childhood malignancies, endocrine late effects, which manifest progressively over the years until well into adulthood, constitute a high burden of disease (6–11). Especially in survivors of CPBTs endocrinopathies are common late effects due to affections of the central endocrine regulating structures of the hypothalamic-pituitary region (12, 13).

Closely related to the endocrine late effects, bone health has been shown to be impaired in a significant number of children and adults surviving pediatric malignancies (14, 15). Adolescent survivors of CPBTs are prone to osteoporosis as reported by Cohen et al. (16) and Remes et al. (17).

To date the onset of osteopathologies in survivors of childhood malignancies is not yet well characterized. Recent studies in ALL indicate that impaired bone health may be observed as early as at the time of manifestation of the malignancy (18, 19) and is present in many survivors during follow-up (15).

Bone health is difficult to assess since a comprehensive set of clinical, biochemical, radiographic and life style factors needs to be analyzed and interpreted, as reviewed recently by Mostoufi-Moab and Ward (20). In the pediatric setting, this assessment is further complicated by the need to apply an age- and pubertal stage-appropriate interpretation of these parameters. Beyond low bone mineral content and alterations in bone metabolism, chronic bone pain, pathological fractures and stunted growth may also account for impaired bone health in pediatric patients with chronic disease including survivors of childhood malignancies (21).

Due to the detrimental effect of endocrine dysfunction (e.g., deficiency of sex steroids) on bone health, and based on the feed-back mechanism between bone metabolism and metabolic health (22, 23) it appears that endocrine late effects, disease comorbidity and bone health are related in survivors of CPBTs, for review see: Desentis-Desentis et al. (24).

In this study we report endocrine status and occurrence of osteopathologies in a single center cohort of 102 survivors of childhood brain malignancy.

## Materials and methods

### Patients

All patients who had been diagnosed with CPBT from 1997 until 2013 and were undergoing follow-up at the oncology outpatient clinic of the tertiary care center from August 2014 until August 2016 were invited to participate in this single-center cross-sectional study.

Detailed data regarding stratification and treatment of the patients are presented in Table 1.

**TABLE 1:** Stratification and treatment of the cohort.

		Number of patients
Gender	Female, Male	47; 55
Mean age at study	13.0 years (SD 4.06, range: 2.39–21.8)	
Mean age at diagnosis	8.66 years (SD 4.13, range: 0.01–17.5)	
Time from diagnosis	4.32 years (SD 3.3, range: 0.22–15.7)	
Diagnosis	Glioma (all types), Medulloblastoma; Ependymoma Germinoma; ATRT; Meningeoma; Craniopharyngeoma; PNET	57; 18; 12 9; 2; 2 1; 1
Localization	Cerebellum; telencephalon; diencephalon; pons; mesencephalon; spinal cord; medulla oblongata	40; 28; 26 3; 2; 2; 1
Hypothalamic-pituitary region	Yes/no	30/72
WHO grading	1/2/3/4	44/14/11/33
Metastases	M0/M1/M2/M3/M4	85/7/5/5/0
Surgical resection	Yes/no	84/18
Residual tumor	Yes/no	34/48
Relapse pre visit	Yes/no/multiple	14/85/3
Irradiation	None/conventionally fractionated photon/ Hyperfractionated photon/proton beam irradiation	48/40 11/1
Chemotherapy	Yes/no	54/48
Combination of treatment modalities	None (watch and wait) Surgery only Chemotherapy only Irradiation only Surgery + Chemotherapy Surgery + Irradiation Chemotherapy + Irradiation Surgery + Chemotherapy + Irradiation	2 34 7 4 6 8 5 36

*Total numbers are displayed for gender, diagnosis, WHO grade, treatment modalities, application of surgical resection, residual tumor at visit, relapse status, (type of) irradiation, application of chemotherapy, and combination of treatment modalities.*

### Clinical parameters and questionnaire

Clinical and anamnestic parameters were assessed as previously described in detail (25): “Briefly, a physical examination, including determination of weight, height and pubertal status was performed. Patients or parents were also asked to complete a standardized questionnaire regarding vitamin D, calcium and nutritional supplement intake, screen hours and hours of physical activity per day. In addition, the patients were asked for presence of bone pain in the form of regularly occurring (on more than half of the days in the last month), spontaneous back pain or exercise related knee pain. Pubertal development was assessed by a pediatric endocrinologist according to Tanner staging. Standard deviation scores (SDS) for pubertal development were calculated using ‘Puberty Plot Web Application’ by Stef van Buuren^1^ (26). The application calculates pubertal SDS for breast development, pubic hair stage and testicular volume based on the Tanner stages for breast development, testicular volume and pubic hair stage. Skeletal age was determined by an experienced pediatric radiologist according to the method of Greulich and Pyle using X-ray images of the left hand (27).”

### Chart review

The following parameters were obtained from the patients’ charts: The tumor localization was categorized into the following seven areas: cerebellum, diencephalon, medulla oblongata, mesencephalon, pons, spinal cord, and telencephalon. In addition, the localization was categorized according to the proximity to the pituitary. Tumors in the sella turcica, basal ganglia, thalamus, bottom of third ventricle and chiasma opticum were labeled as “hypothalamic-pituitary region.” The type of diagnosis including grading were documented. The staging was recorded according to Chang classification for medulloblastoma (M0 = localized, M1 = dissemination of tumor cells to the CSF, M2 = intracranial metastases, M3 = spinal metastases, and M4 = metastases outside of the CNS; 28). The treatment modalities were recorded separately for surgery, chemotherapy and irradiation. In addition, the status of residual tumor and the number of relapses before the study visit were documented. The cumulative dosage of chemotherapy as well as type and dosage of irradiation dosage patient “as treated.”

Upon chart review, fractures which had occurred after the diagnosis of brain tumor were recorded. Vertebral fractures or more than two subsequent fractures of long bones were categorized as pathological fractures.

Endocrinopathies were documented from the patients’ charts if a prior diagnosis of diabetes insipidus, growth hormone (GH) deficiency, precocious puberty, delayed pubertal development, hypocortisolism or a thyroid disorder (Hashimoto thyroiditis, hypothyroidism, and Graves’ disease) was documented.

### Laboratory tests

The following biochemical parameters of growth, pubertal status, bone turnover and vitamin D metabolism were assessed in serum or plasma samples as part of the routine diagnostic laboratory workup in the central laboratory of the University Hospital Essen: 25-OH vitamin D (ng;ml); 1,25-(OH)_2_ vitamin D (pg;ml); serum phosphate (mmol/l), serum calcium (mmol/l), albumin (g/dl), total serum alkaline phosphatase, TSAP (U/l); bone-specific alkaline phosphatase, BAP (U/l); insulin-like growth factor-1, IGF-1 (ng/ml), cortisol (nmol/l), thyroid stimulating hormone, TSH (mU/l), free thyroxine, fT4 (pmol/l), PTH (pg/ml) and osteocalcin (ng/ml). LH (U/L), FSH (U/L), testosterone (nmol/L), estradiol (pg/ml), DHEAS (μg/ml), androstenendione (ng/ml), Inhibin B (ng/l), AMH (μg/l). Plasma levels of osteoprotegerin (pmol/l), receptor activator of nuclear factor kappa-B ligand (RANKL, pmol/l), and tartrate-resistant acid phosphatase 5b (TRAP5b, U/l) were measured using commercially available ELISA assays, according to the manufacturer’s instructions (Osteoprotegerin: OPG, Quidel^®^; RANKL: sRANKL, BIOMEDICA and TRAP 5b: TRAP5b, Quidel^®^; all TECOmedical, Germany).

Serum markers of LH, FSH, testosterone, estradiol, DHEAS, androstenendione, Inhibin B and AMH were measured and interpreted to assess endocrine function in participants, but will not be further discussed in the manuscript.

Additionally, the urinary calcium to creatinine ratio (mg/mg) as well as markers of bone resorption including N-terminal telopeptide, NTX (nmol bone collagen equivalent/mmol creatinine) and deoxypyridinoline, DPD (mg/g creatinine) were assessed in spot urine samples. Pediatric reference ranges were available and applied for all parameters. Serum IGF-1 levels were expressed as SDS values, according to age and sex, based on the data from Blum and Breier (29). To calculate the IGF-1 SDS, we used the software tool “SDSEasy” (Mediagnost, Reutlingen, Germany).

### Bone densitometry

BMD was examined *via* dual-energy X-ray absorptiometry (DXA; Lunar Prodigy, GE-Healthcare, Madison, WI, United States) in a subgroup of patients. BMD was assessed at the lumbar spine (L1 – L4; anteroposterior view) and the left femoral neck. *Z*-scores (DXA Z) were calculated for the lumbar spine measurements based on age specific normal values (30, 31). A single investigator blinded to the clinical status of the patients was responsible for all BMD measurements. Height-adjusted Z-scores (HAZ) from DXA were calculated as described previously (32). The Bone Health Index (BHI) and its SDS (BHI-SDS) was calculated using the software BoneXpert from indices of three metacarpal bones as a parameter to approximate bone density from X-rays of the left hand (33).

### Definition of bone health status

The assessment process of defining the bone health status for the purpose of this study has been previously described in detail (15) The terms “osteopathology” and “impaired bone health” are being used in this manuscript to describe different levels of skeletal late effects. The term “osteopathology” refers to overt bone disease and the term “impaired bone health” refers to a condition with at least one pathological reading in selected parameters of bone health, as displayed in Table 3. Bone health and endocrine status was assessed jointly by two clinical experts (CG and BH). Both of whom are experienced pediatric endocrinologists.

### Vitamin D supplementation

All patients with a vitamin D deficiency (25-OH Vitamin D < 20 ng/ml), as defined by Holick (34) and no signs for decreased renal function or hypercalcemia were started on 1,000 IU of oral Vitamin D3 daily according to the global consensus recommendations (35). 25-OH vitamin D levels were reassessed 3 months later (if possible) and during the regular follow up visit the following year in the survivorship clinic.

### Statistics

Statistical analyses and local polynomial regression fitting was performed with the R base/stats package (36). PRISM for MAC 7.0 (GraphPad Software, Inc., La Jolla, CA, United States) and R base package ggplot2 (37) was used for figures. Values are expressed as the mean ± standard deviation (SD) and range unless stated otherwise. As in most of the variables normal distribution could not be assumed, associations between single variables were described by Spearman correlation coefficient. Differences in continuous variables between the groups were tested using the Mann–Whitney-*U* test for two-group comparisons, and the Kruskal–Wallis tests for more than two groups. When an overall difference was found, *post hoc* analysis was carried out by pairwise comparison of groups using the Mann–Whitney-*U* test, correcting for multiple testing with the Bonferroni-Holm method. Group differences in categorical variables were tested using the chi-square test statistics. For all tests, statistical significance was presumed at *P* < 0.05.

## Results

### Descriptive statistics, endocrine parameters, and parameters of bone health

One hundred four patients were eligible to participate. Of those, two declined for personal reasons. Of the recruited 102 patients, 55 were male, and 47 were female. The majority of patients were diagnosed with glioma of any type (*n* = 57), medulloblastoma (*n* = 18), ependymoma (*n* = 12), and germinoma (*n* = 9). Disease in about half of all patients (*n* = 58) was categorized as WHO grade I and II. The patients were treated with the following modalities: Most patients (*n* = 84) underwent (brain-)surgery and about half of the patients (*n* = 52) underwent irradiation and/or chemotherapy (*n* = 54). The details regarding combination of various treatment modalities are displayed in Table 1.

The mean age of the patients at follow up was 13.0 ± 4.1 years (2.39–21.8). Mean age at diagnosis was 8.66 ± 4.13 years (0.0–17.5), making for a mean of 4.32 ± 3.3 years after first diagnosis of CPBT (range 0.22–15.7).

The mean height SDS was –0.53 ± 1.20 (–4.09–1.86). Mean BMI SDS was 0.72 ± 1.31 (–2.33–4.21). The Tanner stage SDS for pubic hair development and testicular volume/breast development was –0.36 ± 1.00 (–3.02–1.80) and –0.48 ± 1.20 (–3.17–2.01), respectively. In total there were 25 prepubertal (=Tanner 1) children present in this group of survivors.

The questionnaire was completed by 94 of the 102 survivors. The respondents report an average of 2.16 ± 1.53 h (0–5) of daily screen time. Of the patients, eight stated that they would spend most of the day non-ambulatory (sitting or lying down). Fourteen where physically active up to 3 h. Seventy-two were active more than 3 h per day.

More detailed descriptive statistics of the 102 patients are displayed in Table 2 and Supplementary Tables 1A,B. For the following analyses, the applicable age-, sex-, and pubertal status adjusted reference ranges were used for the assessments of the individual data and SDS were applied.

**TABLE 2:** Descriptive statistics of the cohort and parameters of bone health.

	Mean	SD	Min	Max	*n*
*Age (years)*	13.0	4.06	2.39	21.8	102
*Age at Diagnosis (years)*	8.66	4.13	0.01	17.5	102
*Time from initial diagnosis (years)*	4.32	3.30	0.22	15.7	102
*Height-SDS*	–0.53	1.20	–4.09	1.86	97
*Weight-SDS*	0.36	1.39	–3.63	4.33	97
*BMI-SDS*	0.72	1.31	–2.33	4.21	97
*Delta bone age – biological age*	–0.52	1.50	–4.84	2.73	70
*PH-SDS*	–0.36	1.00	–3.02	1.80	62
*TV/BR-SDS*	–0.48	1.20	–3.17	2.01	59
*DXA-Z*	–0.81	1.13	–2.80	2.80	35
*HAZ*	–0.14	0.99	–2.10	2.90	30
*BHI-SDS*	–0.65	1.33	–4.04	2.61	64
*25-OH V D (ng/ml)*	14.7	9.51	2.00	54.3	82
*1,25-(OH)2 V D (pg/ml)*	50.6	20.9	2.50	122.0	83
*PTH (pg/ml)*	42.7	17.7	14.7	107.5	91
*TSAP (U/l)*	196.6	104.4	51.0	622.0	99
*BAP (U/l)*	118.2	71.5	21.0	406.4	97
*Serum-Calcium (mmol/l)*	2.45	0.10	2.23	2.71	99
*Serum-Phosphate (mmol/l)*	1.37	0.23	0.87	2.07	99
*RANKL (pmol/l)*	0.18	0.10	0.00	0.45	87
*OPG (pmol/l)*	4.92	1.09	3.08	7.91	49
*RANKL/OPG (pmol/l/pmol/l)*	0.04	0.03	0.00	0.13	47
*TRAP5b (U/l)*	7.24	4.12	1.40	15.3	32
*NTX (nmolBCE/nmol crea)*	529.1	626.5	23.0	2588.0	23
*DPD (*μ*g/g crea)*	118.6	72.0	12.0	282.0	45
*Ca:Crea (mg/mg)*	0.08	0.08	0.00	0.33	73
*Osteocalcin (ng/ml)*	85.2	35.4	20.9	162.6	35
*IGF-1 SDS*	–0.96	2.10	–9.46	2.14	100
*TSH (mU/l)*	2.14	1.64	0.01	11.7	99
*fT4 (pmol/l)*	14.5	2.18	7.90	20.8	99
*Cortisol (nmol/l)*	274.7	140.6	8.00	719.0	100
*Vitamin D intake (U/d)*	2.48	1.72	0.08	9.03	94
*Calcium intake (mg/d)*	792.7	310.2	122.1	1518.4	94
*Screen-time (h)*	2.16	1.53	0.00	5.00	94

*Mean, SD, range, male adult normal values and the number of patients examined are displayed. SDS, standard deviation score; BMI, body mass index; PH, pubic hair stage; TV/BR, testicular volume/breast development stage; DXA, dual-energy X-ray absorptiometry; HAZ, Height adjusted Z-scores; BHI, Bone Health Index; 25-OH VD, 25-OH vitamin D; 1,25-OH VD, serum 1,25-(OH)2 vitamin D; PTH, parathyroid hormone; TSAP, total serum alkaline phosphatase; BAP, bone-specific alkaline phosphatase; RANKL, Receptor activator of nuclear factor kappa-B ligand; OPG, osteoprotegerin; TRAP5b, tartrate-resistant acid phosphatase 5b; NTX, urinary N-terminal telopeptide; DPD, urinary deoxypyridinoline; Ca:Crea, urinary calcium to creatinine ratio; IGF-1, insulin-like growth factor-1 SDS; TSH, thyroid stimulating hormone; and fT4, free thyroxine.*

**TABLE 3:** Frequency of aberrant findings suggesting impaired bone health.

	Fraction of patients affected	Percentage of patients affected
Elevated PTH	10/91	11.0
Elevated or decreased TSAP/BAP	11/94	11.7
Elevated or decreased DPD/NTX	11/64	17.2
Elevated or decreased Osteocalcin	0/35	0
Decreased urinary Ca excretion	6/74	8.1
Pathological fractures	2/94	2.1
Fractures of long bones	10/94 (2 × 2)	10.6
Low BMD (DXA-Z/HAZ < -2)	5/35	14.3
Bone pain (Knee or Back)	31/93	33.3
Osteonecroses	0/102	0
25-OH VD deficiency (<20 ng/ml)	63/82	76.8
Severe 25-OH VD def. (<10 ng/ml)	31/82	37.8

*Number and percentage of patients affected by altered parameters of calcium metabolism, bone metabolism, or notable clinical findings are displayed. For the assessment of the calcium metabolism, the following parameters were used: decreased urinary calcium excretion (Ca:Crea ratio < 0.01 mg/mg). For the assessment of bone metabolism, the following parameters were used: elevated levels of urinary N-terminal telopeptide (NTX) or urinary deoxypyridinoline (DPD), elevated total serum alkaline phosphatase (TSAP) and/or bone-specific alkaline phosphatase (BSAP) and elevated PTH. Bone-related pain in form of knee pain associated with exercise and recurrent back pain in the last 3 months as well as the history of pathological fractures and fractures of long bones was also included.*

**FIGURE 1:**
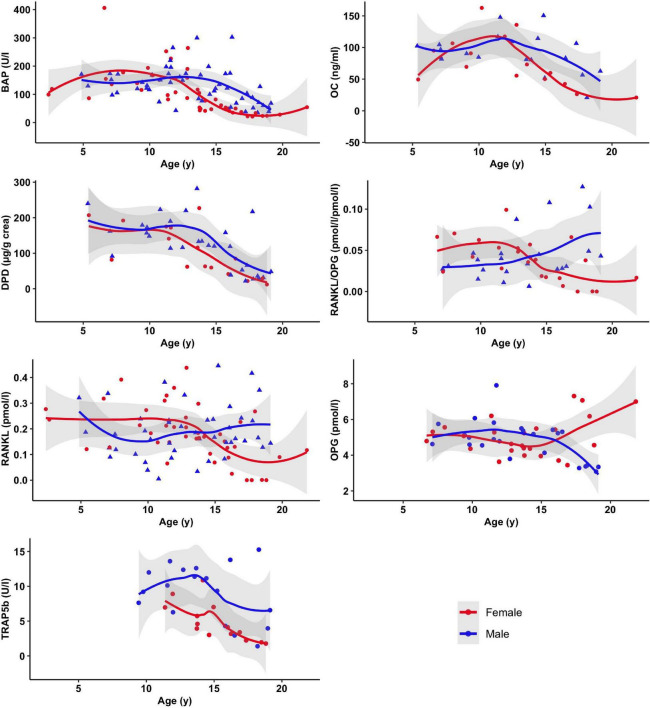
Selected markers of bone metabolism as a function of age and sex. Displayed are mean and 95% confidence band of SE of smoothed curves by local polynomial regression.

As expected, parameters of bone metabolism (BAP, Osteocalcin, DPD, TRAP5B, and Osteoprotegerin) displayed an age and puberty-associated pattern with mostly lower values in the postpubertal age group (Figure 1).

The frequency of aberrant biochemical and radiological findings based on normative values as described in Schündeln et al. (15) are summarized in Table 3. Overall in 65% of patients any kind of bone health impairment was detected. Non-pathological fractures and non-severe vitamin D deficiency were not counted as evidence for impaired bone health. Likewise, the levels of OPG and RANKL were not accounted for in this categorization, since no normative values in childhood and adolescents are available.

### Bone health according to gender

The mean DXA Z score was similar in female and male patients (–0.73 ± 1.45 vs. –0.87 ± 0.90 for both, *P* = 0.95). Similar results were found when comparing the height adjusted *Z*-scores (HAZ, 0.19 ± 1.01 vs. -0.42 ± 0.90, *P* = 0.15). No further difference in bone health related parameters were detectable between male and female patients for the parameters displayed in Table 2.

### Bone health status with regards to disease classification, therapy, and bone metabolism

An expert opinion on the status of bone health was obtained for all patients. Twenty-six patients were labeled as “bone healthy” or “most likely bone healthy” and 28 patients were labeled as affected with “osteopathology” or “most likely affected with osteopathology.” In 48 patients a definite classification based on the available parameters by the experts was not possible. Of these, 32 patients were not categorizable due to unconcise or conflicting findings and data. For 16 patients, data was incomplete.

In univariate analyses, no difference regarding patients’ sex, age or age at diagnosis and the status of bone health was detected. A higher tumor grade than WHO° 1 did not significantly increase the odds ratio for osteopathologies (“osteopathology” or “most likely osteopathology”; Odds ratio = 0.65, 95% CI = 0.22–1.90, *P* = 0.43), nor did the presence of a higher than M0 stage (Odds ratio = 1.5, 95% CI = 0.37–6.60, *P* = 0.57), or the status of relapse (Odds ratio = 0.63, 95% CI = 0.11–3.12, *P* = 0.57) affect bone health status in the cohort. Likewise, the type of treatment was not found to have an impact on the bone health status in a multivariate analysis (irradiation: Odds ratio = 1.58, 95% CI = 0.42–6.58, *P* = 0.50; surgery: Odds ratio = 0.35, 95% CI = 0.04–1.89, *P* = 0.25; and the application of a chemotherapy: Odds ratio = 0.81, 95% CI = 0.19–3.05, *P* = 0.76).

**FIGURE 2:**
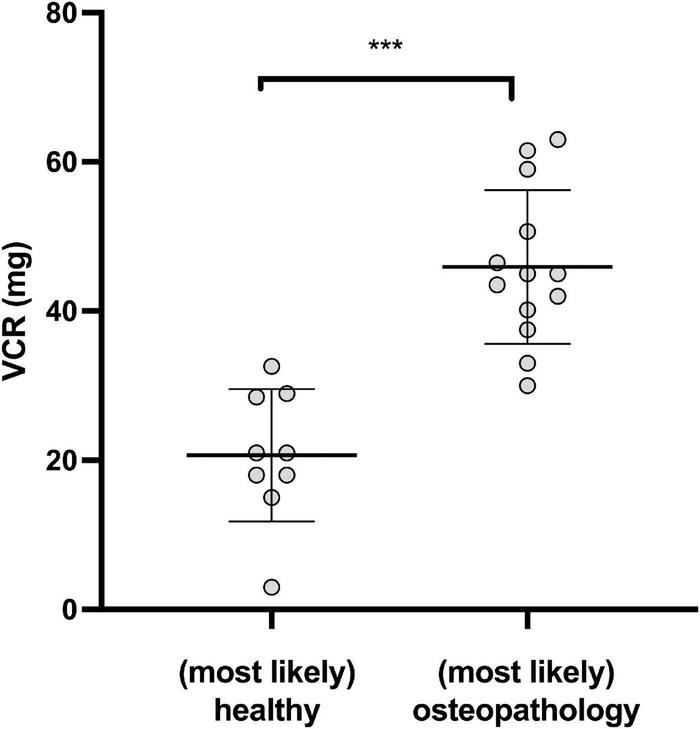
Osteopathologies and Vincristine (VCR). Cumulative dosage of VCR in patients with (most likely) osteopathologies and (most likely) healthy survivors. (***P < 0.001).

However, in a small number of patients, receiving vincristine (VCR), the cumulative dosage was associated with the bone health status: Patients with osteopathologies had received significantly higher amounts of cumulative VCR than patients without osteopathologies [45.9 ± 10.3 (*n* = 9) vs. 20.7 ± 8.88 (*n* = 13) mg/m^2^, *P* < 0.001, Figure 2].

### Bone health and calcium/vitamin D metabolism

**FIGURE 3:**
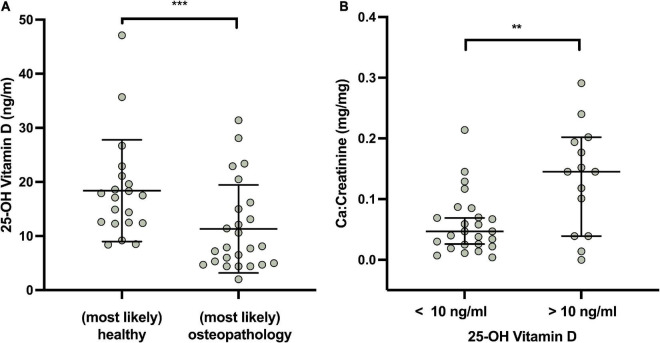
Vitamin D (in ng/ml), osteopathologies and calcium metabolism in survivors. **(A)** Expert opinion and Vitamin D levels. **(B)** Urinary Ca/Creatinine in patients with severe vitamin D deficiency and normal vitamin D values. Lines indicate mean and standard deviation. Statistically significant differences between the groups, determined by Mann–Whitney test, are indicated with asterisks (***P < 0.001; **P < 0.01).

Vitamin D deficiency serum 25 OH vitamin *D* < 20 ng/ml, as defined by the Institute of Medicine (38) was found in 76.8% of the patients and a severe vitamin D deficiency [serum 25 OH vitamin *D* < 10 ng/ml (34)] in 37.8% of patients. Figure 3 shows a lower overall vitamin D level in patients with osteopathologies (11.3 ± 8.14 vs. 18.4 ± 9.39 mg/mg, *P* < 0.05). In the group of patients with sufficient vitamin D levels the calcium:creatinine ratio in urine was higher, indicating adequate calcium stores, compared to the group of patients who displayed a severe vitamin D deficiency (0.13 ± 0.09 vs. 0.06 ± 0.05 mg/mg, *P* = 0.01, Figure 3).

**FIGURE 4:**
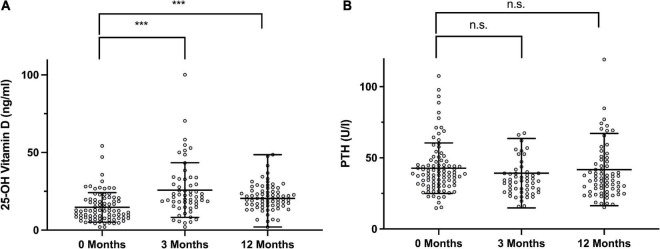
Longitudinal Follow Up of 25-hydroxy vitamin D and PTH levels. **(A)** Vitamin D at the initial visit, after 3 months and after 12 months (+12 M). **(B)** Parathyroid hormone (PTH) at the respective time points. Statistically significant differences between the groups, determined by Mann–Whitney test, are indicated with asterisks (***P < 0.001).

Following supplementation, a significant increase in vitamin D levels 14.7 ± 9.51 ng/ml to 25.8 ± 17.6 ng/ml (*P* < 0.001) was observed after 3 months, however, this effect waned slightly to a mean of 21.2 ± 8.77 ng/ml after 12 months, which, nonetheless was significantly higher than the initial value (*P* < 0.001, Figure 4). The levels of PTH decreased accordingly, although not significantly from 42.7 ± 17.7 ng/ml to 39.2 ± 24.4 ng/ml after 3 months to 41.7 ± 25.4 ng/ml after 1 year.

### Endocrine late effects and bone health

The assessment of survivors for diagnosed pathologies of the endocrine system is summarized in Table 4. In brief, 10.8% of patients had a diagnosis of diabetes insipidus centralis due to loss of anti-diuretic hormone (ADH) production, 13.7% showed aberrant pubertal development, in most cases due to a central hypogonadism with low gonadotropins. A central hypocortisolism based on low ACTH production, resulting in secondary adrenal insufficiency, was present in 14.9%. GH deficiency was diagnosed in 21.8%. A thyroid dysfunction was found in 23.8% of the survivors, again mostly based on central hypothyroidism with impaired TSH production. Each of the central deficiencies (ADH, ACTH, Gonadotropins, TSH and GH) is described as one “endocrine axis” in the following text and figures.

**TABLE 4:** Endocrine health as diagnosed at initial visit.

Pituitary axis/specific diagnosis	*n*	Not impaired [fraction (%)]	Impaired [fraction (%)]
**Posterior pituitary** Diabetes insipidus	102	91 (89.2)	11 (10.8) 11
**Hypothalamo-pituitary-gonadal axis**	102	88 (86.3)	14 (13.7)
Central hypogonadism			8
Hypergonadotropic hypogonadism			1
Precocious puberty			5
**Hypothalamo-pituitary-adrenal axis** Secondary adrenal insufficiency	101	86 (85.1)	15 (14.9) 15
**Hypothalamo-pituitary-IGF-1 axis**	101	79 (78.2)	22 (21.8)
GH deficiency			21
Short stature and low IGF-1			1
**Hypothalamo-pituitary-thyroid axis**	101	77 (76.2)	24 (23.8)
Central hypothyroidism			23
Autoimmune thyroiditis			1

*Total number of patients, fraction and percentage (applicable for specific diagnosis), fraction (percentage) categorized “not impaired” (applicable for pituitary axes), and fraction (percentage) categorized “impaired” (applicable for endocrine axes) are displayed for each examined endocrine axis, followed by specific diagnoses referring to that axis.*

Patients with endocrine late effects were treated and monitored according to international standards of care for their endocrine diseases by pediatric endocrinologists of the university hospital.

There was a cumulative effect of irradiation on affected endocrine axes in the study cohort. In summary, 31.3% of survivors displayed an endocrinopathy in at least one axis. In 21.6% more than one axis was involved. The fraction of survivors with at least one axis involved was higher in irradiated patients than in non-irradiated patients (40.1 vs. 12.2%).

The localization of the tumor was significantly related with endocrine function. Patients with a tumor categorized as “located near hypothalamic structures” had a 3.7 times higher likelihood of one or more impaired axes compared to the rest of the cohort (Odds ratio = 3.69, 95% CI = 1.52–9.26, < *0.01*).

In a multivariate analysis, irradiation, but not chemotherapy or surgery in a patient was associated with a higher likelihood for (at least one) impaired endocrine axis (irradiation*:* Odds ratio = 8.53, 95% CI = 2.80–30.6, *P* < 0.001; chemotherapy*:* Odds ratio = 1.37, 95% CI = 0.45–4.04, *P* = 0.42; and surgery*:* Odds ratio = 0.88, 95% CI = 0.26–3.06, *P* = 0.83).

Patients with high grade (WHO grade III or IV) CPBT had a higher number of impaired axes (*P* < 0.01 in WHO°I and°II CPBT (*n* = 0–4, median = 0) when compared to patients with WHO° III and°IV CPBT (*n* = 0–5, median = 0).

Patients with one or more affected axes were more prone to develop (most likely) osteopathologies (79% with osteopathologies vs. 21% without osteopathologies, *P* < 0.01).

**FIGURE 5:**
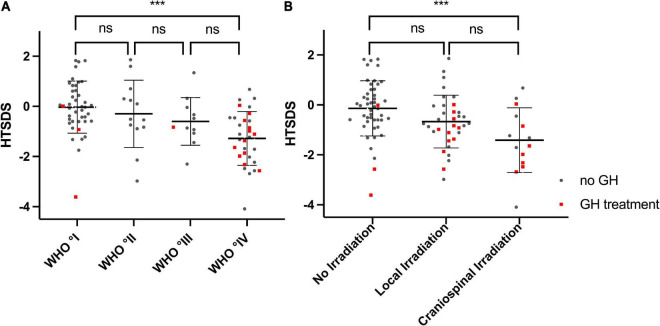
Height SDS and WHO Degree, Height SDS and irradiation treatment: **(A)** Height SDS is decreasing with increasing WHO degree of the tumors (*P* < 0.001 Kruskal–Wallis test). **(B)** Height SDS is lower in patients who received irradiation (*P* < 0.001 Kruskal–Wallis test), likely due to the increased percentage of patients with a GH deficiency in that group. Lines indicate mean and sd. Red dots represent individuals who receive(d) GH treatment. ***p < 0.001.

In the analyzed cohort, the survivors’ height SDS was correlated inversely with the WHO° of tumors. (Figure 5A, *P* < 0.001, Kruskal–Wallis test). Patients with WHO° IV tumors displayed significantly lower height SDS compared to patients diagnosed with WHO° I tumors [–1.28 ± 1.07 vs –0.03 ± 1.04, (*P* < 0.001, Mann–Whitney-*U* test with Bonferroni-Holm correction for multiple testing)]. In addition, survivors who had undergone irradiation therapy, were of shorter stature than non-irradiated individuals (Figure 5B, *P* < 0.001). After correcting for multiple testing a significant difference was seen between between non-irradiated patients and patients with craniospinal irradiation [–1.41 ± 1.30 vs –0.14 ± 1.10, (*P* < 0.001)].

## Discussion

In survivors of childhood malignancies, endocrine late effects and osteopathologies constitute late effects with a high burden of disease (39). In many aspects this is especially relevant following the diagnosis of CPBT. With a focus on endocrine and bone health, this study from a large German tertiary center describes late effects of 102 adolescent survivors after diagnosis of CPBT.

In the present cohort, about two third of survivors displayed impaired bone health with at least one pathological reading of the assessed parameters. Especially (secondary) hyperparathyroidism, abnormal findings in alkaline phosphatase levels as well as in the bone resorption markers NTX and DPD were prevalent. Clinically, one third of the patients were experiencing bone pain regularly.

However, punctual pathological findings of one or some of the many parameters of bone health may not reflect overt osteopathology. We therefore attempted to further categorize the bone health status of survivors with or without osteopathologies. A categorization was only possible in about half of the cohort. In some cases, information was missing and in many cases data was unconcise. This was comparable to our experience with the data in survivors of childhood ALL (15) and reflects the complexity and difficulty of the task to identify those survivors in need for attention for bone health issues in the routine clinic [as recently reviewed by Marcucci et al. (14)].

Regarding parameters of bone health, we hypothesized that survivors suffering from endocrine late effects, and survivors with more aggressive tumors and higher intensity of treatment or irradiation would be more prone to osteopathologies. However, we were not able to pinpoint any specific factors leading to osteopathologies with respect to tumor entity, stage of tumor or treatment modality, with the exception of higher vincristine (VCR) dosages (see below). This may be a sign of robustness or resilience of the childhood bone remodeling process (40). Nonetheless, we did find a positive association of VCR dose and osteopathologies in the subgroup of patients who had been treated with VCR. A possible causal explanation could be a VCR associated neuropathy leading to decreased physical activity and thus impaired bone health. Additionally, a VCR-related disturbance in sodium regulation may lead to osteopenia (41, 42). However, other unidentified confounding factors may be present.

The hormone vitamin D plays an important role in the maintenance of calcium homeostasis and thereby bone health but may have extraskeletal effects aswell (43, 44). Severe vitamin D deficiency in the present cohort was associated with lower calcium stores, as indicated by low calcium excretion in the urine, and with osteopathologies. The fraction of survivors with severe Vitamin D deficiency was higher than reported for the general German population (45). Preventative measures, including avoidance of sun exposure and a higher rate of immobility/in-house activity may contribute to lower levels of vitamin D in these children. Additionally, in this cohort of CPBT survivors a high proportion of recent immigrants/refugees are present, a group which is known to be more prone to be vitamin D deficient in Germany (46). Supplementation of vitamin D_3_ did lead to a significant increase of serum levels initially. During follow-up, however, as reported by others as well (47) adherence waned off and PTH and vitamin D serum levels after 1 year were similar to the initial values. These results highlight the need for regular follow up with information of doctors, nurses and families on the importance of sufficient vitamin D stores and support of survivors. Screen time in the group of survivors was similar to that in the general pediatric population, according to a recent study by Schmidt et al. (48).

In this study, the height SDS of survivors was inversely correlated to the WHO degree of the tumor. And, like other groups (49) we could show that growth was more stunted in children who received irradiation. Irradiated patients also displayed a higher number of impaired endocrine axes, pointing toward the intertwined effects of irradiation, the resulting hormonal deficiencies and the tumor stage on growth in children and adolescents. As shown before (50, 51), patients who underwent irradiation or chemotherapy were prone to develop central endocrine dysfunction [as also reviewed by Roddy and Mueller (52)]. It proved impossible to disentangle the effects of therapy on the hormonal system (e.g., GH axis or gonadotropic-gonadal axis) from effects on bone metabolism.

In the present cohort, endocrine late effects with impairment of at least one endocrine axis was detected in one third of the survivors and impairment of more than one axis was found in one fourth of the cohort. This is a higher rate of endocrine late effects than expected and described by other authors and is likely explained by the comprehensive analyses and thorough assessment of hormonal parameters. Of note, endocrine deficiencies may be underdiagnosed in pediatric patients with brain tumors, due to subtle clinical signs with potentially multifactorial causes and the challenging biochemical workup. This conclusion is shared by other authors (12). A recommendation for regular follow-ups of survivors of CPBTs with pediatric endocrinology even in the absence of overt hormonal deficiencies seems warranted. As described previously by Shaw (53) patients with tumors in proximity to the hypothalamic-pituitary region were more likely to display hormonal deficiencies. The extend of endocrine disease, expressed as number of impaired axes, did not correlate with the presence of diagnosed osteopathologies.

## Limitations

The goal of this study was to thoroughly describe bone status and endocrine function of a large group of CPBT survivors. It has, however, several limitations. The cohort is heterogeneous regarding type and grade of tumor, treatment modality, etc., which makes it difficult to draw conclusions from a cross-sectional study, even though the cohort is representative of the spectrum of CPBTs typically seen in a large tertiary center. A multicentric approach with longitudinal follow-up would be desirable and may lead to more concise results. Also, with a cross sectional study such as this one, unlike a longitudinal study (12), one can only make limited assumptions regarding the time course of the described findings.

The high rate of survivors with a undeterminable bone health status is comparable to the rate in the previously described cohort of ALL patients. This – again – mirrors the aforementioned challenges to reliably describe bone health.

## Conclusion

This study indicates that endocrine deficiencies are common and (maybe) underdiagnosed in pediatric survivors of CPBTs. Osteopathologies are present in this cohort, however, at a lower rate than in survivors of ALL. A prominent effect of hormonal deficiencies on bone health was not detected, possibly indicating that patients were either sufficiently treated for the endocrine conditions or being a sign of robustness of the childhood bone remodeling process. Vitamin D should be supplemented as recommended. Sustained supplementation and patient adherence are laborious and hard to accomplish.

## Data Availability Statement

The raw data supporting the conclusions of this article will be made available by the authors, without undue reservation.

## Ethics statement

The studies involving human participants were reviewed and approved by Research Ethics Committee of the Medical Faculty, University of Duisburg-Essen. Written informed consent to participate in this study was provided by the participants or their legal guardian/next of kin and eligible patients.

## Author contributions

CG, MS, and BH: study conception and design. SF, SG, PH, MS, CK, and MM: acquisition of data. BH, CG, MS, and SF: analysis and interpretation of data. MS, CG, and SF: drafting and revising the manuscript. All authors read and approved the final manuscript.

## Conflict of Interest

The authors declare that the research was conducted in the absence of any commercial or financial relationships that could be construed as a potential conflict of interest.

## Publisher’s Note

All claims expressed in this article are solely those of the authors and do not necessarily represent those of their affiliated organizations, or those of the publisher, the editors and the reviewers. Any product that may be evaluated in this article, or claim that may be made by its manufacturer, is not guaranteed or endorsed by the publisher.

## Footnotes

1 http://vps.stefvanbuuren.nl/puberty/, accessed, March 28, 2022

## Abbreviations

ACTH: Adrenocorticotrophic hormone
ADH: Antidiuretic hormone
25-OH VD: Serum levels of 25-OH vitamin D
BSAP: Bone-specific alkaline phosphatase
BMI: Body mass index
Ca:crea: Urinary calcium to creatinine ratio
CPBT: Childhood primary brain tumor
DPD: Urinary deoxypyridinoline
GH: Growth Hormone
HAZ: Height-adjusted Z-scores
IGF-1: Insulin-like growth factor-1
NTX: Urinary N-telopeptide
PTH: Parathyroid hormone
RANKL: Receptor activator of nuclear factor kappa-B ligand
SD: Standard deviation
SDS: Standard deviation score
TRAP5b: Tartrate-resistant acid phosphatase 5b
TSAP: Total serum alkaline phosphatase
TSH: Thyroid-stimulating hormone
VCR: Vincristine
