# Supplementary material for: Prevalence of osteopathologies in a single center cohort of survivors of childhood primary brain tumor
Source: Front Pediatr. 2022 Jul 18;10:913343. doi: 10.3389/fped.2022.913343 (PMC9339690; doi:10.3389/fped.2022.913343)
Supplement: Supplementary file 1 [file Table_1.docx]

***Supplemental Table 1a,***

*Descriptive statistics of prepubertal patients (Tanner Stage I) at time of study-visit,*

|  | mean | sd | min | max | n |
| --- | --- | --- | --- | --- | --- |
| *Age (years)* | **7,87** | 2,71 | 2,39 | 12,87 | 25 |
| *Age at Diagnosis (years)* | **5,09** | 3,34 | 0,01 | 11,05 | 25 |
| *Time from initial diagnosis (years)* | **2,78** | 2,62 | 0,22 | 9,38 | 25 |
| *Height-SDS* | **-0,31** | 1,23 | -2,57 | 1,78 | 24 |
| *Weight-SDS* | **0,71** | 1,01 | -0,97 | 2,68 | 24 |
| *BMI-SDS* | **1,10** | 1,03 | -0,80 | 2,88 | 24 |
| *Delta bone age - biological age* | **-0,58** | 1,49 | -4,29 | 2,73 | 18 |
| *PH-SDS* | **-0,56** | 0,87 | -1,93 | 0,48 | 6 |
| *TV/BR-SDS* | **-1,05** | 0,73 | -2,14 | -0,33 | 6 |
| *DXA-Z* | **-0,10** | 0,75 | -0,90 | 0,60 | 3 |
| *HAZ* | **-0,30** | 0,26 | -0,50 | 0,00 | 3 |
| *BHI-SDS* | **-0,69** | 1,34 | -4,04 | 1,38 | 16 |
| *25-OH V D (ng/ml)* | **12,08** | 6,39 | 2,00 | 28,30 | 20 |
| *1,25-(OH)2 V D (pg/ml)* | **51,95** | 21,35 | 26,00 | 100,00 | 19 |
| *PTH (pg/ml)* | **43,13** | 18,30 | 22,80 | 97,80 | 24 |
| *TSAP (U/l)* | **253,76** | 111,27 | 98,00 | 622,00 | 25 |
| *BAP (U/l)* | **161,03** | 70,48 | 86,20 | 406,40 | 23 |
| *Serum-Calcium (mmol/l)* | **2,47** | 0,09 | 2,28 | 2,61 | 25 |
| *Serum-Phosphate (mmol/l)* | **1,54** | 0,20 | 1,23 | 2,00 | 25 |
| *RANKL (pmol/l)* | **0,20** | 0,11 | 0,04 | 0,44 | 22 |
| *OPG (pmol/l)* | **5,31** | 1,02 | 4,36 | 7,91 | 10 |
| *RANKL/OPG (pmol/l/pmol/l)* | **0,04** | 0,02 | 0,01 | 0,07 | 10 |
| *TRAP5b (U/l)* | **10,62** | 4,23 | 7,63 | 13,60 | 2 |
| *NTX (nmolBCE/nmol crea)* | **736,40** | 470,96 | 275,00 | 1506,00 | 5 |
| *DPD (µg/g crea)* | **161,54** | 48,81 | 81,70 | 240,00 | 10 |
| *Ca:Crea (mg/mg)* | **0,09** | 0,09 | 0,00 | 0,33 | 15 |
| *Osteocalcin (ng/ml)* | **87,73** | 16,90 | 49,50 | 106,60 | 11 |
| *IGF-1 SDS* | **-0,12** | 1,32 | -3,21 | 2,14 | 24 |
| *TSH (mU/l)* | **2,71** | 1,42 | 0,01 | 5,29 | 24 |
| *fT4 (pmol/l)* | **14,23** | 2,07 | 9,80 | 17,40 | 24 |
| *Cortisol (nmol/l)* | **272,21** | 82,05 | 141,00 | 440,00 | 24 |
| *Vitamin D intake (U/d)* | **2,85** | 2,29 | 0,35 | 9,03 | 24 |
| *Calcium intake (mg/d)* | **784,69** | 262,91 | 165,00 | 1174,29 | 24 |
| *Screen-time (h)* | **1,63** | 1,13 | 0,00 | 4,00 | 24 |

|  | mean | sd | min | max | n |
| --- | --- | --- | --- | --- | --- |
| *Age (years)* | **15,03** | 2,85 | 9,07 | 21,83 | 61 |
| *Age at Diagnosis (years)* | **9,91** | 3,93 | 1,70 | 17,46 | 61 |
| *Time from initial diagnosis (years)* | **5,12** | 3,44 | 0,32 | 15,66 | 61 |
| *Height-SDS* | **-0,75** | 1,18 | -4,09 | 1,86 | 57 |
| *Weight-SDS* | **0,27** | 1,61 | -3,63 | 4,33 | 57 |
| *BMI-SDS* | **0,71** | 1,43 | -2,33 | 4,21 | 57 |
| *Delta bone age - biological age* | **-0,66** | 1,52 | -4,84 | 2,29 | 46 |
| *PH-SDS* | **-0,34** | 1,02 | -3,02 | 1,80 | 56 |
| *TV/BR-SDS* | **-0,41** | 1,23 | -3,17 | 2,01 | 53 |
| *DXA-Z* | **-0,88** | 1,21 | -2,80 | 2,80 | 28 |
| *HAZ* | **-0,02** | 1,02 | -2,00 | 2,90 | 24 |
| *BHI-SDS* | **-0,70** | 1,34 | -3,36 | 2,61 | 43 |
| *25-OH V D (ng/ml)* | **15,72** | 10,74 | 2,00 | 54,30 | 52 |
| *1,25-(OH)2 V D (pg/ml)* | **51,43** | 20,85 | 11,00 | 122,00 | 51 |
| *PTH (pg/ml)* | **43,84** | 18,81 | 14,70 | 107,50 | 55 |
| *TSAP (U/l)* | **171,49** | 92,88 | 51,00 | 439,00 | 59 |
| *BAP (U/l)* | **100,97** | 64,96 | 21,00 | 302,70 | 60 |
| *Serum-Calcium (mmol/l)* | **2,44** | 0,11 | 2,23 | 2,71 | 59 |
| *Serum-Phosphate (mmol/l)* | **1,31** | 0,23 | 0,87 | 2,07 | 59 |
| *RANKL (pmol/l)* | **0,18** | 0,10 | 0,00 | 0,45 | 53 |
| *OPG (pmol/l)* | **4,86** | 1,11 | 3,08 | 7,31 | 36 |
| *RANKL/OPG (pmol/l/pmol/l)* | **0,04** | 0,03 | 0,00 | 0,13 | 34 |
| *TRAP5b (U/l)* | **7,06** | 4,21 | 1,40 | 15,26 | 27 |
| *NTX (nmolBCE/nmol crea)* | **440,62** | 721,81 | 23,00 | 2588,00 | 13 |
| *DPD (µg/g crea)* | **99,73** | 72,64 | 12,00 | 282,00 | 29 |
| *Ca:Crea (mg/mg)* | **0,09** | 0,07 | 0,00 | 0,29 | 47 |
| *Osteocalcin (ng/ml)* | **80,68** | 39,97 | 20,90 | 150,40 | 20 |
| *IGF-1 SDS* | **-1,38** | 2,40 | -9,46 | 1,98 | 61 |
| *TSH (mU/l)* | **1,96** | 1,80 | 0,01 | 11,72 | 60 |
| *fT4 (pmol/l)* | **14,60** | 2,26 | 7,90 | 20,80 | 60 |
| *Cortisol (nmol/l)* | **276,20** | 156,41 | 8,00 | 684,00 | 61 |
| *Vitamin D intake (U/d)* | **2,44** | 1,48 | 0,08 | 6,84 | 58 |
| *Calcium intake (mg/d)* | **764,96** | 320,89 | 122,14 | 1518,43 | 58 |
| *Screen-time (h)* | **2,48** | 1,59 | 0,00 | 5,00 | 58 |

***Supplemental Table 1b,***

*Descriptive statistics of patients with Tanner Stage II-V at time of study-visit,*
